# Preoperative risk factors including serum levels of potassium, sodium, and creatinine for early mortality after open abdominal surgery: a retrospective cohort study

**DOI:** 10.1186/s12893-021-01070-0

**Published:** 2021-01-26

**Authors:** Mohamed Ebrahim, Pia Bükmann Larsen, Damoun Hannani, Sara Liest, Lars Nannestad Jørgensen, Henrik Løvendahl Jørgensen

**Affiliations:** 1 Digestive Disease Center, Bispebjerg Hospital, University of Copenhagen, 2400 Copenhagen, NV Denmark; 2 Department of Clinical Biochemistry, Hvidovre Hospital, University of Copenhagen, Copenhagen, Denmark; 3 Department of Clinical Medicine, University of Copenhagen, Copenhagen, Denmark

**Keywords:** Electrolytes, Creatinine, Mortality, Surgery, Algorithm, Frailty

## Abstract

**Background:**

In hospitalized patients, abnormal plasma electrolyte concentrations are frequent and have been linked to poor outcomes following acute surgery. The aim of this study was to assess whether preoperative plasma levels of potassium, sodium, and creatinine at the time of admission were associated with 30-day mortality in patients following open abdominal surgery.

**Methods:**

This was a single-center register-based retrospective study. By means of electronic search in a maintained surgery database, all patients (n = 4177) aged ≥ 60 years old undergoing open surgery in our department from January 2000 to May 2013 were identified. Plasma was assessed within 30 days prior to surgery. The primary endpoint was 30-day postoperative mortality. The association between mortality and plasma levels of potassium, sodium, and creatinine were examined using Cox proportional hazard models.

**Results:**

A total of 3690 patients were included in the study cohort. The rates of abnormal preoperative plasma levels were 36, 41, and 38% for potassium, sodium, and creatinine, respectively. The overall 30 day mortality was 20%. A predictive algorithm for 30 day mortality following abdominal surgery was constructed by means of logistic regression showing excellent distinction between patients with and without a fatal postoperative outcome.

**Conclusion:**

Apart from demographic factors (age, sex, and emergency surgery), preoperative imbalance in potassium, sodium and creatinine levels were significant independent predictors of early mortality following open abdominal surgery.

## Background

With the increasing proportion of elderly in the Western world, there is a rise in patients needing surgical care including emergency abdominal surgery [1, 2]. While acute abdominal surgery can be lifesaving, it carries a considerable risk of postoperative morbidity and mortality [3–6]. Early mortality following open emergent abdominal surgery ranges between 12 and 20% with an overrepresentation of elderly patients [3–15]. These patients often have comorbidities, reduced physiologic reserves, or frailty making them susceptible to complications and death [3–5, 7, 10–13, 16].

In order to identify patients at a high risk of postoperative complications and poor outcomes, different scoring systems have been proposed [11, 13–15, 17]. However, most of these require as many as 15–18 variables, e.g. the APACHE II and the P-POSSUM scoring systems, thus limiting their use in the acute setting [18, 19].

Physiologic electrolyte rearrangements are frequently observed in hospitalized patients [20]. However, little is known about preoperative electrolyte levels and the risk of postoperative mortality following open abdominal surgery [15, 21–24]. The aim of this study was therefore to examine if preoperative levels of potassium, sodium, and creatinine were associated with 30-day mortality in adults aged ≥ 60 years undergoing open abdominal surgery including elective and emergency surgery. Secondly, the aim was to develop a predictive algorithm for 30-day mortality.

## Methods

### Patients

We extracted data from the Danish National Patient Registry on all open abdominal surgical interventions performed at the Digestive Disease Center, Bispebjerg Hospital, Denmark, on patients aged ≥ 60 years during the period from January 1st, 2000 to May 31st, 2013, n = 4177.

A total of 81 different types of surgery according to the NOMESCO Classification of Surgical Procedures were grouped into seven categories depending on the anatomical location of the procedure (Additional file 1: Table S1).

Only the primary surgical procedure (The index operation) was assessed.

**Fig. 1:**
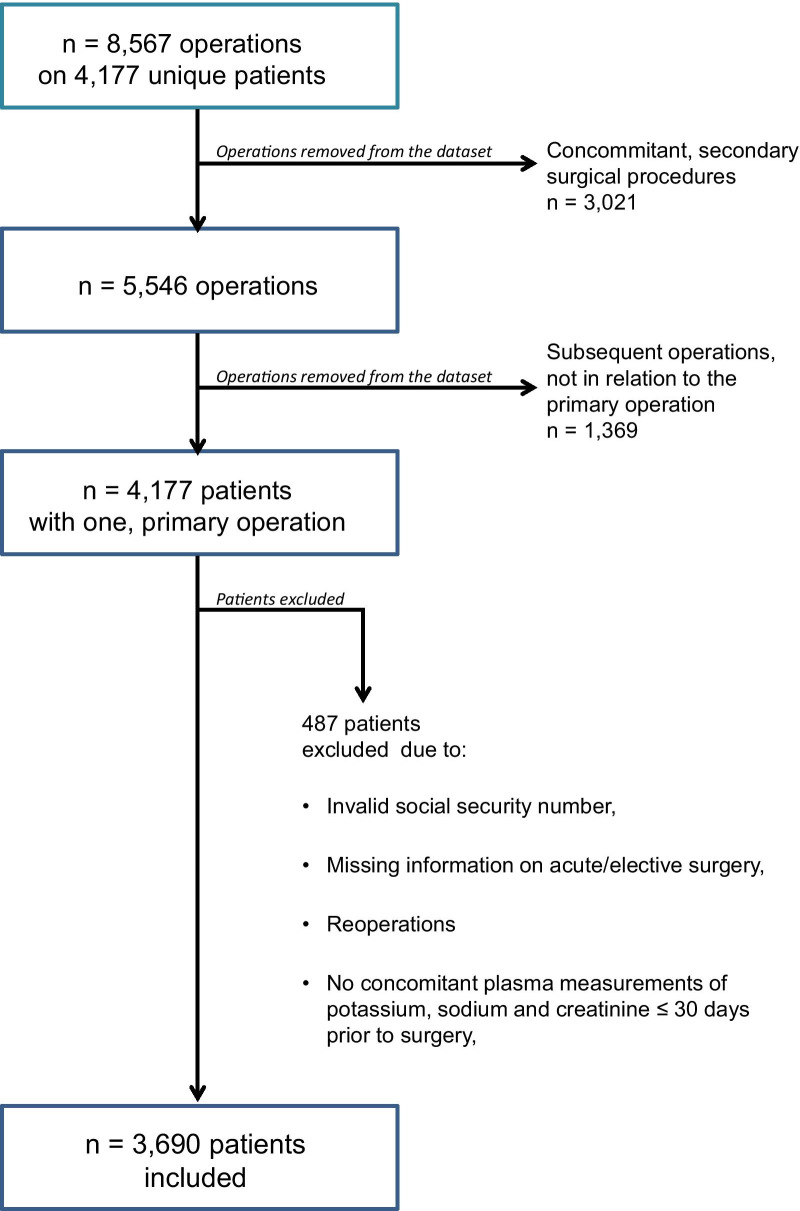
Flowchart of the patient selection process. “4177 unique patients with 8567 operations were assessed. Only the primary index operation was assessed thus 3021 concomitant secondary surgical operations were removed from the dataset (e.g. If a patient underwent a colonic resection and concomitant adhesiolysis, the adhesiolysis procedure was not assessed). 1369 subsequent operations not in relation to the primary operation were removed from the dataset (e.g. subsequent appendectomy years after a cholecystectomy). We thus had 4177 patients with one unique operation each. 487 patients were excluded due to the reasons given in Fig. 1. This left 3690 patients for analysis”

Patients with a temporary civil registration number, missing information regarding surgical priority or lacking concomitant plasma measurements of potassium, sodium, and creatinine within 30 days prior to the surgical intervention were excluded. This left 3690 patients for analysis. The selection process is described in detail in Fig. 1.

From the civil registration system, we extracted data on vital status and emigration. Using the unique civil registration number which all Danish citizens have, the data from the three registries were merged into a single anonymized database used for data analysis in this study.

Open abdominal surgery was defined as surgical procedure involving incision through the abdominal wall (laparotomy) to facilitate access into the abdominal cavity.

Elective surgery was defined as surgical procedures scheduled in advance to admission while acute surgery was defined as urgent surgical procedures due to an acute illness posing threat to life or tissue.

### Biochemical measurements

Results of plasma measurements of potassium, sodium, and creatinine measurements were extracted from the local laboratory information system. In case of more than one biochemical measurement being present within 30 days prior to the surgical intervention, the one closest to the date of the surgery was used.

P-potassium, P-sodium and P-creatinine were all measured at the Department of Clinical Biochemistry at Bispebjerg Hospital and throughout the study period, the department took part in external quality assurance programs assuring stable levels. The normal ranges of the biochemical markers were 3.5—4.4 mmol/L for P-potassium, 137—144 mmol/L for P-sodium, 60—105 micromole/L (males) and 50—90 micromole/L (females) for P-creatinine.

### Statistical analyses

The primary endpoint was 30-day mortality.

Differences between continuous and categorical variables were tested using Mann–Whitney *U* tests and the chi-square tests, respectively. Logistic regression analysis was used to test the association between the preoperative biochemical markers and 30-day mortality with results presented as odds ratios (OR) with 95% confidence intervals (CI). The analyses were either unadjusted or multivariably adjusted for the relevant covariates.

A stepwise multivariate logistic regression analysis was carried out and variables were removed one at a time from the model if p-values were greater than 0.05, starting with the least significant variable. The results of the stepwise logistic regression analysis were used to assess the probability of death within 30 days after surgery (Pd30):

$$\ln \left( {\frac{{Pd30}}{{1 - Pd30}}} \right) = intercept + estimate1 \times covariate1 + estimate2 \times covariate2 + \ldots$$

After isolation of Pd30 in this equation, the probability of death within 30 days after surgery can be estimated in a prediction model (Additional file 2):

$$Pd30 = \frac{{exp\left( {intercept + estimate1*covariate1 + estimate2*covariate2 + \ldots } \right)}}{{\left( {1 + exp\left( {\left( {intercept + estimate1*covariate1 + estimate2*covariate2 + \ldots } \right)} \right)} \right)}}$$

$$Multiple\, logistic\, regression\, equation\, for\, the\, final\, model\,(\mathrm{S})$$

$$S= -9.6827+0.4446*hyperK+0.365*hypoK+1.1397*hyperNa+0.4705*hypoNa+0.0076*creatinine+1.5767*acute+0.2324*sex+0.0564*age+1.7488*group1+1.2143*group3+1.4724*group5+1.4164*group6+1.3708*group7$$

$$Pd30\left(\%\right)=\frac{100*{e}^{S}}{1+{e}^{S}}$$

Receiver Operating Characteristics (ROC) was used to compare the relation between sensitivity and specificity for the prediction model, for the individual biochemical markers and for age. The area under the curve (AUC) was calculated for each parameter to compare their discriminative abilities. Differences between the AUCs were tested using the general linear model.

All analyses were carried out using the SAS statistical software package (SAS Institute, Cary, NC). The statistical level of significance was defined as *p* < 0.05.

### Approvals

Permission for this study was granted by the Danish Data Protection Agency (ID no. 2007–58-0015). No permission from the regional Ethics Committee was needed since the study was purely registry-based.

The study was reported according to The Strengthening the Reporting of Observational Studies in Epidemiology (STROBE) guidelines [25].

## Results

**Fig. 2:**
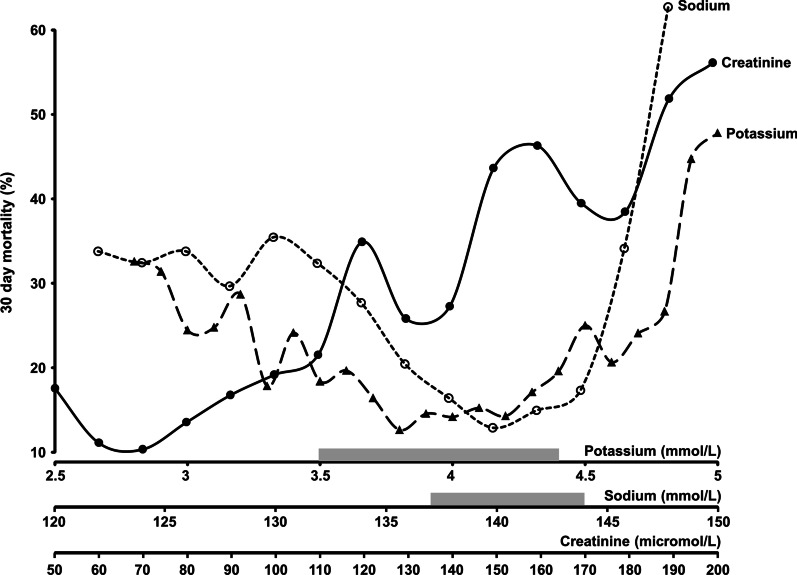
30-day mortality by plasma levels of potassium, sodium, and creatinine. P-Potassium was rounded to the nearest value divisible by 0.1. Values ≤ 2.8 mmol/L were set at 2.8 mmol/L. Values ≥ 5 mmol/L were set at 5 mmol/L. P-Sodium was rounded to the nearest value divisible by 2. Values ≤ 122 mmol/L were set at 122 mmol/L. Values ≥ 148 mmol/L were set at 148 mmol/L. P-Creatinine was rounded to the nearest value divisible by 10. Values ≤ 50 micromole/L were set at 50 micromole/L. Values ≥ 200 micromole/L were set at 200 micromole/L. For P-Potassium and P-Sodium, the normal ranges were indicated by grey boxes on their respective axes

A total of 3690 patients with a median age of 76.8 years were included in the study. Most of the patients (61.5%) were females. Overall, the rates of patients within the normal ranges were 64.2% for potassium, 59.2% for sodium, and 62.2% for creatinine. The 30 day mortality rates in the entire study population, the elective group and the emergency group were 20.1, 5.3 and 27.4% respectively. The basic characteristics of the patients by vital status 30 days postoperatively are presented in Table 1. In Fig. 2, unadjusted 30 days mortality according to the levels of the three-biochemical markers is shown. For potassium and sodium, the patients within the normal ranges had the lowest mortality compared to the patients both below and above the normal ranges whereas mortality increased linearly with increasing levels of creatinine. Tables 2, 3, 4 show the odds ratios (OR) for 30 days mortality with increasing levels of adjustment for P-potassium, P-sodium, and P-creatinine, respectively. In Table 5, the result of the stepwise logistic regression including all three biochemical markers as well as all the available covariates is shown. Potassium and sodium were entered categorically into the analysis as within, below, or above the normal ranges due to their non-linear relationship with 30 days mortality found in Fig. 2. Time from blood test to surgery and surgical group IV (appendix) did not reach statistical significance and were therefore excluded from the final model. As an example, a 65 year old male undergoing acute surgery of the small bowel with the following biochemical values: P-Potassium 5.4 mmol/L, P-Sodium: 135 mmol/L, and P-Creatinine: 121 micromole/L would have a 23.9%-risk of dying with 30 days after surgery.

**Table 1:** Basic characteristics

	Alive after 30 days	Dead within 30 days	*P*-value
N (%)	2950	740	–
Age (years)^*^	75.2 (67.7; 82.2)	81.7 (75.1; 86.9)	< 0.0001
Females, N (%) / males N (%)^**^	1806 (61) / 1144 (39)	463 (63) / 277 (37)	0.5
Time from blood test to surgery (hours)^*^	20.5 (7.3; 27.2)	8.8 (4.2; 19.0)	< 0.0001
Acute surgery, N (%) / eletive surgery, N (%)^**^	1792 (61) / 1158 (39)	675 (91) / 65 (9)	< 0.0001
P-Sodium (mmol/L)^*^	138 (135; 140)	137 (133; 140)	< 0.0001
P-Potassium (mmol/L)^*^	3.9 (3.5; 4.2)	3.8 (3.4; 4.3)	0.8
P-Creatinine (micromol/L)^*^	81 (67; 100)	102 (79; 152)	< 0.0001
Surgical category, N (%)^**^			
I. Gastroduodenal	226 (8)	136 (18)	
II. Biliary	199 (7)	15 (2)	
III. Small bowel	216 (7)	75 (10)	
IV. Appendix	162 (6)	16 (2)	< 0.0001
V. Colon	1156 (39)	270 (37)	
VI. Rectum	447 (15)	49 (7)	
VII. Herniotomy, adhesiolysis, splenectomy, and diagnostic purposes	544 (18)	179 (24)	

”*”Medians and interquartile range (IQR) are shown. Differences were tested using Mann–Whitney U tests

“**”Numbers and percentages are shown. Differences were tested using chi-square statistics

**Table 2:** Potassium

	Unadjusted	Adjusted for age and sex	Fully adjusted^†^
Hyperkalemia (N = 466)	2.41 (1.93; 3.02)****	2.22 (1.76; 2.80)****	2.08 (1.62; 2.66)****
Hypokalemia (N = 855)	1.80 (1.49; 2.17)****	1.71 (1.40; 2.08)****	1.38 (1.13; 1.69)**
Age (per 10 years)	–	2.01 (1.82; 2.22)****	1.80 (1.62; 1.99)****
Male vs female	–	1.28 (1.07; 1.54)**	1.42 (1.18; 1.72)***
Time from blood test to surgery (per hour)	–	–	1.00 (1.00; 1.00)
Acute vs eletive surgery	–	–	5.39 (3.98; 7.31)****
I. Gastroduodenal (N = 362)	–	–	5.69 (3.17; 10.22)****
III. Small bowel (N = 291)	–	–	3.26 (1.78; 5.98)****
IV. Appendix (N = 178)	–	–	1.11 (0.52; 2.36)
V. Colon (N = 1426)	–	–	3.9 (2.23; 6.82)****
VI. Rectum (N = 496)	–	–	3.53 (1.87; 6.69)****
VII. Herniotomy, adhesiolysis, splenectomy, and diagnostic purposes (N = 723)	–	–	3.99 (2.26; 7.04)****

Odds ratios and 95% confidence intervals for 30-day mortality. Biliary surgery (N = 214) and normokalaemia (N = 2369) were used as reference for their respective groups

^†^Adjusted for age, sex, surgical priority( elective vs. emergency) and type of surgery(I-VII)

****P < 0.0001, ***P < 0.001, **P < 0.01

**Table 3:** Sodium

	Unadjusted	Adjusted for age and sex	Fully adjusted^†^
Hypernatremia (N = 140)	4.41 (3.09; 6.28)****	4.03 (2.78; 5.84)****	3.53 (2.38; 5.23)****
Hyponatremia (N = 1364)	2.12 (1.79; 2.51)****	2.12 (1.78; 2.52)****	1.61 (1.34; 1.93)****
Age (per 10 years)	–	2.02 (1.83; 2.24)****	1.82 (1.64; 2.01)****
Male vs female	–	1.34 (1.12; 1.60)**	1.47 (1.21; 1.77)****
Time from blood test to surgery (per hour)	–	–	1.00 (1.00; 1.00)
Acute vs eletive surgery	–	–	1.59 (1.29; 1.89)****
I. Gastroduodenal (N = 362)	–	–	5.79 (3.23; 10.41)****
III. Small bowel (N = 291)	–	–	3.24 (1.77; 5.95)****
IV. Appendix (N = 178)	–	–	1.08 (0.51; 2.29)
V. Colon (N = 1426)	–	–	3.91 (2.23; 6.85)****
VI. Rectum (N = 496)	–	–	3.48 (1.83; 6.60)****
VII. Herniotomy, adhesiolysis, splenectomy, and diagnostic purposes (N = 723)	–	–	4.01 (2.27; 7.10)****

Odds ratios and 95% confidence intervals for 30 day mortality. Biliary surgery (N = 214) and normonatremia (N = 2186) were used as reference for their respective groups

^†^Adjusted for age, sex, surgical priority( elective vs. emergency) and type of surgery(I-VII)

****P < 0.0001, ***P < 0.001, **P < 0.01

**Table 4:** Creatinine

	Unadjusted	Adjusted for age and sex	Fully adjusted^†^
Creatinine (per 10 micromol/L)	1.11 (1.09; 1.13)****	1.10 (1.08; 1.12)****	1.08 (1.07; 1.10)****
Age (per 10 years)	–	1.92 (1.73; 2.12)****	1.74 (1.56; 1.92)****
Male vs female	–	1.04 (0.86; 1.25)	1.19 (0.98; 1.44)
Time from blood test to surgery (per hour)	–	–	1.00 (1.00; 1.00)
Acute vs eletive surgery	–	–	1.62 (1.31; 1.92)****
I. Gastroduodenal (N = 362)	–	–	6.00 (3.32; 10.86)****
III. Small bowel (N = 291)	–	–	3.32 (1.79; 6.14)****
IV. Appendix (N = 178)	–	–	1.14 (0.53; 2.43)
V. Colon (N = 1426)	–	–	4.37 (2.47; 7.71)****
VI. Rectum (N = 496)	–	–	4.03 (2.11; 7.69)****
VII. Herniotomy, adhesiolysis, splenectomy, and diagnostic purposes (N = 723)	-	-	3.92 (2.20; 6.99)****

Odds ratios and 95% confidence intervals for 30 day mortality. Biliary surgery (N = 214) was used as reference

^†^Adjusted for age, sex, surgical priority ( elective vs. emergency) and type of surgery(I-VII)

****P < 0.0001, ***P < 0.001, **P < 0.01

**Table 5:** Final model

	OR	Estimate	P-value
Intercept	–	− 9.6827	< 0.0001
Creatinine (OR per 10 micromol/L)	1.08 (1.06; 1.10)	0.0076	< 0.0001
Hyperkalemia (N = 466)	1.56 (1.20; 2.03)	0.4446	0.001
Hypokalemia (N = 855)	1.44 (1.17; 1.78)	0.3650	0.0006
Hypernatremia (N = 140)	3.13 (2.08; 4.70)	1.1397	< 0.0001
Hyponatremia (N = 1364)	1.60 (1.32; 1.93)	0.4705	< 0.0001
Age (OR per 10 years)	1.76 (1.58; 1.96)	0.5640	< 0.0001
Male vs female	1.26 (1.04; 1.54)	0.2324	0.02
Acute vs eletive surgery	4.84 (3.58; 6.54)	1.5767	< 0.0001
I. Gastroduodenal (N = 362)	5.75 (3.15; 10.48)	1.7488	< 0.0001
III. Small bowel (N = 291)	3.37 (1.81; 6.27)	1.2143	< 0.0001
V. Colon (N = 1426)	4.36 (2.45; 7.75)	1.4724	< 0.0001
VI. Rectum (N = 496)	4.12 (2.14; 7.94)	1.4164	< 0.0001
VII. Herniotomy, adhesiolysis, splenectomy, and diagnostic purposes (N = 723)	3.94 (2.20; 7.07)	1.3708	< 0.0001

Odds ratios and 95% confidence intervals for 30 day mortality, *P*-values and estimates from the logit function for the variables reaching significance after stepwise logistic regression analysis

Biliary surgery (N = 214), Normokalemia (N = 2369) and Normonatremia (N = 2186) are used as reference for their respective groups

**Fig. 3:**
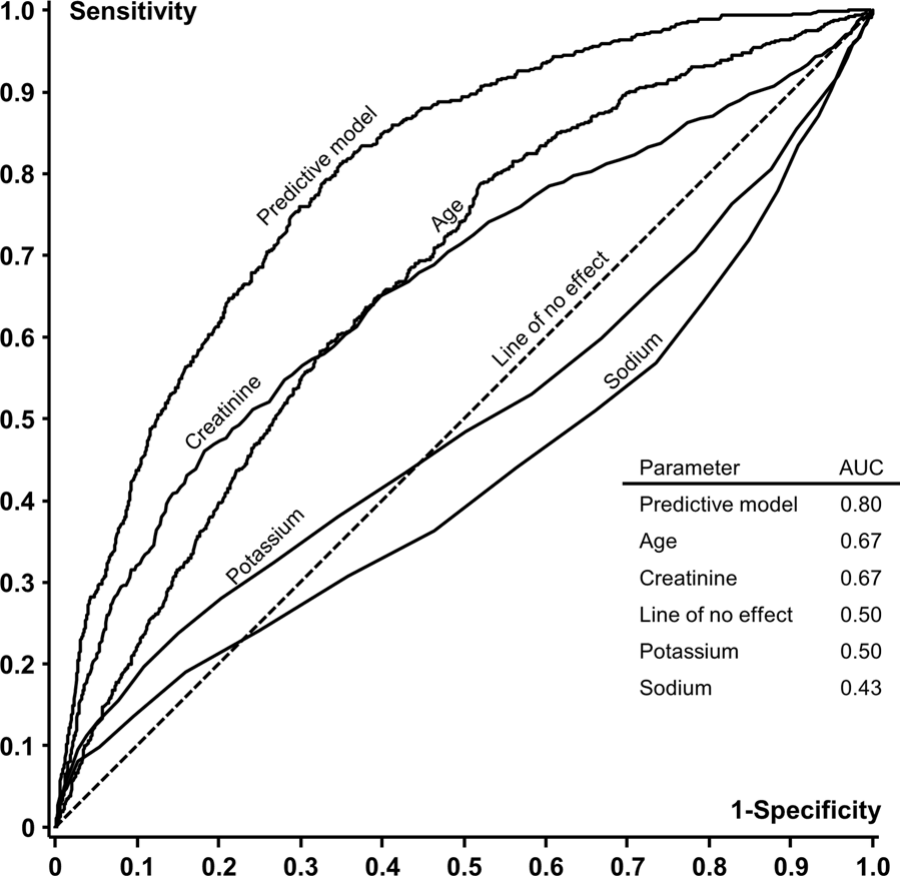
Receiver Operating Characteristics (ROC) for the predictive model, age, P-potassium, P-sodium, and P-creatinine. *AUC* area under the curve

In Fig. 3, ROC curves for the predictive model, age, creatinine, potassium, and sodium are shown. The predictive model had the highest area under the curve, 0.80, compared to age, potassium, sodium, and creatinine individually. (P < 0.0001). Due to the non-linear relationship of 30 days mortality with potassium and sodium, the curves for these two parameters cross the line of no effect.

## Sensitivity analyses

The patients undergoing acute surgery had a considerably higher 30-days mortality rate than those undergoing elective surgery (Table 5). In order to assess whether acute versus elective surgery was the major decisive factor in the model, we calculated a modified model using only the 1223 patients undergoing elective surgery. This model still had an AUC of 0.75 for the prediction of 30 days mortality superior to the AUCs of age, creatinine, potassium, and sodium individually.

A total of 352 patients were excluded due to missing concomitant plasma measurements of potassium, sodium and creatinine ≤ 30 days prior to surgery (the remaining 135 patients of the total of 487 not included in the study were excluded due to one of the other three points in Fig. 1). These 352 excluded patients were younger than the included 3690 patients, 70.4 years versus 76.1 years (*P* = 0.0001). The gender distribution was similar with males accounting for 40.1% of the excluded patients vs 38.5% of the included patients (*p* = 0.6) and they had a lower rate of acute surgery 47.9% vs 66.9% (*p* < 0.0001). Finally, the 30 days mortality was lower 9.4 vS 20.1% (*p* < 0.0001). This might have led to a slight overestimation of the effect of acute surgery in the prediction model.

## Discussion

This study shows a clear positive association between disturbance in preoperative levels of potassium, sodium, and creatinine and 30 days mortality following open abdominal surgery in patients aged ≥ 60 years. Our findings are in line with previous findings from patients undergoing orthopedic surgery [26–29]. However, little is published about preoperative electrolyte and creatinine levels and mortality risk following open abdominal surgery [21–24].

The 27%-30 day mortality rate following open emergency surgery revealed in this study was higher than 8–20% reported from other centres [4–6]. Contrary to these studies, our patient population was older and did not undergo minimal invasive procedures. Patients operated on by laparoscopy were not included in our study because this technique was adopted during the study period. Inclusion of such might have biased our results due to bias from a surgical learning curve. Recent developments in minimally invasive surgery—including placement of colorectal stents in patients with large bowel obstruction as a bridge to elective surgery—have reduced surgical trauma during emergency conditions. Moreover, access to intensive care, awareness, and standardization of care for patients undergoing emergency surgery have improved. Taken together, these developments have led to lower postoperative mortality [30].

Emergent abdominal surgery among elderly patients is frequent and associated with significant morbidity and mortality posing a considerable clinical challenge. Appropriate risk assessment is a key concern to reconcile treatment purpose and expectations when advising patients and their relatives. Different prediction systems have been proposed to identify patients at high risk of poor outcomes following emergency abdominal surgery. Many of these prediction tools including the APACHE II and the P-POSSUM scoring systems require as many as 18 variables and are time-consuming thus limiting their clinical use [18, 19].

In real-world practice, preoperative decision making is often based on the surgeon’s subjective opinion, which may vary by experience. Our proposed predictive model for 30 days mortality, which includes potassium, sodium, and creatinine levels, risk factors such as age, sex, and type of surgery may aid in preoperative risk stratification. Furthermore, results from preoperative blood tests are readily available and require only limited costs. Pre-existing electrolyte and creatinine abnormalities are common in the aging population due to functional changes in the renal system including a decrease in glomerular filtration rate and decreased capability of electrolyte excretion [20]. Moreover, comorbidities and polypharmacy may also have an impact on pre-operative electrolyte and fluid status making the patient vulnerable to an acute surgical stress response. When these patients face an acute surgical illness i.e., gastrointestinal fluid loss due to hemorrhage or vomiting due to bowel obstruction, impairment of the physiological system, and limited compensatory mechanisms due to reduced physiologic reserves may promote complications.

Taken together, our findings and those of previous studies indicate that frail patients need to be identified properly through a multidisciplinary approach including geriatric assessment. This may be the subject of future studies to decrease morbidity and mortality. Treatment goals should carefully be elicited and addressed early in the patient assessment to optimize patient care and select the patients who may benefit from surgery [31, 32].

While the main strength of the current study is the high number of patients included, certain limitations should be taken into consideration when the results of the current study are interpreted. Firstly, this is a single-center retrospective exploratory cohort study and due to the nature of the study design, the findings may not be generalizable to other centers. As we did not have access to medical records and only focused on biochemical data (preoperative concentration of potassium, sodium, and creatinine), we could not distinguish between reversible and nonreversible morbidities in the included patients. Also, we did not have reliable information on the cause of death nor information on comorbidities that may have had an impact on mortality following surgery. We also did not include potentially confounding factors like perioperative complications, perioperative fluid administration, blood loss, operative time, and the surgeons experience.

Finally, we cannot draw a conclusion about the external validity of our model as it has not yet been validated in an external prospective cohort representing other hospitals or countries and in patients subjected to minimally invasive surgery.

## Conclusion

Apart from well-established risk factors (male sex, advanced age and emergency surgery), elevated plasma levels of potassium, sodium and creatinine in elderly patients undergoing open abdominal surgery were predictive of increased 30-day mortality. Although a cause-effect relationship cannot be determined from these data, the proposed 30-day mortality prediction algorithm was reliable in our exploratory study. However, the algorithm needs to be validated in a prospective cohort to finally define its clinical suitability.

## Supplementary Information

**Additional file 1: Table S1** Type of surgery performed.

**Additional file 2.** Predictive model for 30 days mortality in in patients undergoing open abdominal surgery.

## Data Availability

The data that support the findings of this study are available on request from the corresponding author. However, the data are not publicly available by The Danish Health Authority since they contain information that may compromise participant privacy.

## Abbreviations

APACHE II: Acute Physiology and Chronic Health Evaluation II
P-POSSUM: Portsmouth Physiological and Operative Severity Score for the Enumeration of Mortality
NOMESCO: The Nordic Medico-Statistical Committee
