# Supplementary material for: Preoperative risk factors including serum levels of potassium, sodium, and creatinine for early mortality after open abdominal surgery: a retrospective cohort study
Source: BMC Surg. 2021 Jan 26;21:62. doi: 10.1186/s12893-021-01070-0 (PMC7836189; doi:10.1186/s12893-021-01070-0)
Supplement: Supplementary file 1 — Additional file 1: Table S1 Type of surgery performed. [file 12893_2021_1070_MOESM1_ESM.docx]

**Supplementary Table 1** Type of surgery performed

| Group number | Operation type |
| --- | --- |
| Group I | Gastroduodenal surgery due to peptic ulcer repair including resections with or without anastomosis |
| Group II | Biliary surgery mainly cholecystectomy |
| Group III | Small bowel surgery including stoma formation and small bowel resections |
| Group IV | Appendectomy |
| Group V | Colonic resections with or without stoma formation |
| Group VI | Rectal resections with or without stoma formation |
| Group VII | Herniotomy, adhesiolysis, splenectomy, and diagnostic purposes |
